# Rectal ulceration caused by the anti-anginal nicorandil: Case report of a preventable complication

**DOI:** 10.1186/1754-9493-4-10

**Published:** 2010-06-30

**Authors:** Sachin Malde, Ajay Wilson

**Affiliations:** 1 Department of Urology, Wythenshawe Hospital, Southmoor Road, Manchester, M23 9LT, UK; 2 Department of Surgery, Stepping Hill Hospital, Poplar Grove, Stockport, SK2 7JE, UK

## Abstract

The association of the anti-anginal drug nicorandil with oral and anal ulceration is becoming more widely recognised, but there are no reports of isolated nicorandil-induced rectal ulceration. Awareness of this condition is poor, and patients often undergo unnecessary surgery for a condition which resolves on stopping the medication.

We report a case of nicorandil-induced rectal ulceration causing rectal bleeding. The patient was spared surgery after awareness of the link with this drug.

This diagnosis should be considered in patients with unexplained gastrointestinal ulceration after exclusion of serious underlying causes. We hope this report will increase awareness amongst physicians and surgeons of this reversible condition.

## Background

Nicorandil, a nicotinamide ester, was originally introduced in Japan in 1984 and subsequently launched in Europe ten years later. Acting as both a venodilator and arteriolar vasodilator, it reduces cardiac preload and afterload, and increases coronary blood flow [1]. It is widely used as a second-line medication in the prevention and long-term treatment of angina. In 2002 a landmark randomised controlled trial, the IONA study, showed that as well as providing symptomatic benefit nicorandil also had cardioprotective properties [2]. Since then, it has been widely prescribed and is particularly useful in patients with refractory or unstable angina.

Common side-effects of nicorandil therapy include headaches, flushing, nausea and dizziness, but more recently a number of reports have linked its use with oral [3], anal [4], vulval and parastomal ulceration [5], as well as anal fistulation [6] (see Appendix 1). However, there are currently no reports in the English literature linking nicorandil therapy with ulceration of the rectum. Furthermore, there is still a lack of awareness of nicorandil-induced ulceration, and consequently this diagnosis is often overlooked. We present a case of rectal bleeding secondary to nicorandil-induced rectal ulceration and aim to increase awareness of this reversible condition.

### Case presentation

An 80 year-old man with a background history of prostate cancer, requiring radiotherapy in 2003, was admitted under the surgical team in 2009 with recurrent episodes of fresh rectal bleeding which began 3 years earlier. He had numerous admissions since 2006, and investigation with flexible sigmoidoscopy and an abdominal CT scan at that time showed multiple telangiectasia and diverticular disease. He was diagnosed with radiation proctitis and underwent argon beam therapy However, this failed to improve his symptoms and in 2008 he developed worsening rectal bleeding. He had been advised to try steroid enemas, but this didn't have any effect.

He had a history of severe ischaemic heart disease for which he had a coronary artery bypass graft, and after a subsequent myocardial infarction underwent coronary angioplasty and stent insertion. Despite this, he continued to have angina, and so was started on nicorandil 10 mg twice daily in 2006. Of note, in 2008, his nicorandil dose was increased to 30 mg twice daily, and it was a few months after this that his rectal bleeding worsened.

He underwent examination under anaesthesia and flexible sigmoidoscopy. This revealed a large area of ulceration with well-defined margins on the anterior rectal wall. Biopsies were taken and histology showed non-specific inflammation with no evidence of malignancy.

In view of the ongoing bleeding, he was listed for abdomino-perineal excision of the rectum, but we became aware of the link with nicorandil, and so the surgery was deferred. The nicorandil was discontinued after discussion with a cardiologist, and over the following weeks the patient's symptoms resolved. The ulceration healed completely and has not recurred since. He remains under close cardiology follow-up to ensure there is no deterioration in his anginal symptoms.

## Discussion

Rectal bleeding is a common surgical complaint and has a multitude of causes. Any patient presenting with this symptom should be fully investigated to identify the underlying cause. Ulceration of the rectal mucosa may cause bleeding, and itself may be the result of various disease processes including malignant and inflammatory conditions. Treatment will vary widely depending upon the cause and so biopsy of any ulceration is essential to aid diagnosis and therefore treatment. More serious underlying causes for rectal ulceration should be excluded before implicating nicorandil.

Reports of nicorandil-induced ulceration first appeared in the French literature in 1997 [7], and since then the number of identified cases has increased. Oral and anal ulceration has been well described, but more recently associations with the gastrointestinal tract have documented. Nicorandil-induced perforation of the terminal ileum has been reported [8], as well as recent reports of multiple synchronous colonic ulcers either in isolation or in combination with anal ulcers [9]. Oesophageal ulceration has also been reported [10] suggesting that any part of the GI tract can be involved. Rectal ulceration, however, has never been reported. As in our case, ulcers are typically described as large punched-out lesions which characteristically show complete resolution on discontinuation of the drug, and biopsy will show non-specific inflammation only. They may occur as early as 2 months after starting nicorandil therapy, but have even been reported 36 months later [11].

The mechanism by which it causes ulceration is not completely understood but suggestions include a direct local effect of nicorandil or one of its metabolites, or a 'vascular steal phenomenon'. A vascular steal phenomenon occurs in vascular beds in which one portion is supplied by a patent artery and the other by a stenosed artery, in a setting of generalised vasodilatation. As the stenosed artery is already maximally dilated in compensation for the distal narrowing, use of vasodilatory drugs will cause blood to flow preferentially to the patent artery, resulting in ischaemia in the territory supplied by the stenosed artery. An interesting feature of this particular case is the fact that the patient has chronic radiation proctitis. Radiation damage results in focal destruction of arterioles, intimal fibrosis and mucosal ischaemia. It is likely, therefore, that the use of nicorandil caused a reduction in blood flow to the areas affected by radiation damage due to a vascular steal phenomenon, resulting in further mucosal ischaemia and subsequent ulceration. The fact that only the rectum was involved in this case further supports this putative mechanism, suggesting that patients with radiation proctitis are at an increased risk of nicorandil-induced ulceration. This should be borne in mind when prescribing this drug.

Whatever the mechanism, it has been suggested that the effect is dose-dependent. The majority of cases are reported in patients taking 40-60 mg daily and it has been found that reducing the dose can improve ulcer healing [12]. Despite this, there have been reports of ulceration in patients taking 10 mg/day [13]. Although treatment with colchicine, thalidomide and prednisolone has been tried [11], ulcers frequently recur. The only definitive treatment is discontinuation of the drug, and complete ulcer healing can take anywhere between 2 weeks to 10 months. This should only be done under supervision by a cardiologist, however, as these patients are at high-risk of recurrence of anginal symptoms. The Medicines and Healthcare Products Regulatory Agency emphasises this point, and patients should remain under close cardiology follow-up to ensure there is no deterioration in the patient's anginal symptoms. If it cannot be discontinued, reducing the dose may improve ulcer healing sufficiently to ease symptoms.

The importance of recognising this condition is that it is reversible upon stopping the medication, and will avoid unnecessary surgery in patients with significant co-morbidities. There have been reports of patients undergoing diversion colostomies for ulceration [14], but the ulcers often recur. In our case, the patient had been listed for abdomino-perineal resection of the rectum, but was spared this major surgery after we became aware of the possible link with nicorandil. The value of a good history cannot be overemphasised. Our patient was repeatedly diagnosed with radiation proctitis given his past history. On careful questioning however, it was found that his symptoms worsened a few months after starting high-dose nicorandil therapy. This eventually led to the diagnosis in this case.

## Conclusion

Nicorandil therapy is becoming increasingly recognised as a cause of gastrointestinal ulceration and it should always be considered in the differential diagnosis of this condition. Ulceration resolves completely on withdrawal of the drug, although this should only be done in conjunction with a cardiologist. Awareness of this condition will prevent unnecessary and major surgery in this high-risk group of patients.

## Consent

Written informed consent was obtained from the patient for publication of this case report and any accompanying images. A copy of the written consent is available for review by the Editor-in-Chief of this journal.

## Competing interests

All authors declare that they have no competing interests, and have no relations to any manufacturer producing the drug described in this study, or to any industrial company which may produce a competing pharmacological agent.

## Authors' contributions

AW had the original idea for the case and managed the patient. SM and AW were involved in reviewing the literature. SM wrote the paper and is guarantor. All authors read and approved the final manuscript.

## Appendix 1

Reported side-effects of nicorandil by likelihood of occurrence

Very common (affect 1 in 10 people)

Headaches

Common (affect between 1 in 10 to 1 in 100 people)

Dizziness

Nausea and vomiting

Flushing of the skin

Uncommon (affect between 1 in 100 and 1 in 1000 people)

Low blood pressure

Increased heart rate

Mouth ulcers

Pain in your muscles

Angioedema

Rare (affect between 1 in 1000 and 1 in 10,000 people)

Skin rashes

Abnormal liver function causing jaundice

Very rare (affect less than 1 in 10,000 people)

Ulcers- Stomach, colon, rectum, anus, genital tract, nasal passages, peri-stomal
